# Conference on Tuberculosis of the Lungs

**Published:** 1919-01

**Authors:** 


### CONFERENCE ON TUBERCULOSIS OF THE LUNGS

In this number we print the report on the Conference on Tuberculosis of the Lungs, which took place at the November meeting of the Research Society of the American Red Cross in France. In spite of the topic being so time-worn, the meeting proved to be one of the best attended and most interesting of the series. In the American Army, an experiment on a large scale was undertaken to exclude soldiers with manifest pulmonary tuberculosis. The discussions at the meeting would indicate that this has met with a large measure of success. That these expert examinations would detect all cases of pulmonary tuberculosis which would break out in the American Expeditionary Forces could hardly be expected. For many years authorities in tuberculosis have been inclined to criticise general practitioners for not recognizing this disease earlier. It is still an open question to-day, even after all the experiences in the Army, whether this very early diagnosis of pulmonary tuberculosis is really possible. The difficulties in differential diagnosis are well brought out, and an analysis of these would strongly suggest that if the sputum is repeatedly negative to tubercle, the chances probably are that one is dealing with a non-tuberculous chest case.

Last spring, on account of the difficulties in diagnosis, the Chief Surgeon of the American Expeditionary Forces very wisely made a ruling that only cases with tubercle bacillus in the sputum should be diagnosed as cases of pulmonary tuberculosis; all other suspected cases should be classified Tuberculosis Observation. Three Centers were selected (Base Hospitals, N$^{os}$ 20, 3 and 8) where these cases for observation could be more thoroughly studied. About the same time the Surgeon-General decided that men should not be discharged from the Army unless the tubercle bacillus was present in the sputum, and that in the United States all other suspected cases should be kept under observation.

Many of the pathologists of the American Expeditionary Forces have taken the opportunities offered them in the autopsies on our young adult soldiers of searching for every possible trace of tubercle. The work reported of Captains Ernst and Opie is of the highest possible merit, and may lead to very important consequences. The discovery that so very many of our young soldiers contain absolutely no discoverable lesions of tubercle is also of the greatest importance, and would at least suggest that we must not accept the fact that, in all classes of people, every one is infected by the time the age of puberty is reached.

We have long known that children with adenoids may have persistent coughs; but, as Major Rist correctly says, the fact that so many adults have nasal affections which cause chronic chest conditions and raise the suspicion of pulmonary tuberculosis are, is a lesson of the war. That broncho-pneumonias do not disappear as text-books have led us to think, but that they may leave considerable residual fractions which disappear very slowly, and are apt to be mistaken for pulmonary tuberculosis, are also important lessons.

Our Government has very wisely established a series of new sanatoria at home for the care of all tuberculous cases. The tuberculous soldiers are obliged to remain in these institutions at least three months. They are given every opportunity to learn how to care for themselves in the future and also to learn new trades while they are taking the cure.

The war efforts of the United States have very greatly assisted in solving the tuberculous problems of the future, and there can be no doubt that, in addition to the scientific advances that have been made in this disease, the attempts at the detection and the care of tuberculous members of communities will result in important social changes.

